# High-Risk PNPLA3 rs738409 Genotype Is Associated with Higher Concentrations of CCL2 in Liver Transplant Candidates with Alcoholic End-Stage Liver Disease

**DOI:** 10.3390/medicina61071293

**Published:** 2025-07-18

**Authors:** Ivan Budimir Bekan, Dino Šisl, Alan Šućur, Ana Bainrauch, Valerija Bralić Lang, Pavao Planinić, Nataša Kovačić, Danka Grčević, Anna Mrzljak, Tomislav Kelava

**Affiliations:** 1 Department of Surgery, Merkur University Hospital, 10000 Zagreb, Croatia; ivan.bekan@gmail.com; 2 Laboratory for Molecular Immunology, Croatian Institute for Brain Research, University of Zagreb, 10000 Zagreb, Croatia; dino.sisl22@gmail.com (D.Š.); alan.sucur@mef.hr (A.Š.); natasa.kovacic@mef.hr (N.K.); danka.grcevic@mef.hr (D.G.); 3 Department of Physiology and Immunology, School of Medicine, University of Zagreb, 10000 Zagreb, Croatia; 4 Department of Internal Medicine, Merkur University Hospital, 10000 Zagreb, Croatia; ana.bainrauch@gmail.com; 5 Private Family Physician Office Zagreb, 10000 Zagreb, Croatia; valerija.bralic@mef.hr; 6 Department of Family Medicine, School of Medicine, University of Zagreb, 10000 Zagreb, Croatia; 7 Department of Physiology, School of Medicine, University of Mostar, 88000 Mostar, Bosnia and Herzegovina; pavaoplaninic98@gmail.com; 8 Department of Anatomy, School of Medicine, University of Zagreb, 10000 Zagreb, Croatia; 9 School of Medicine, University of Zagreb, 10000 Zagreb, Croatia; 10 Department of Gastroenterology and Hepatology, University Hospital Center Zagreb, University of Zagreb, 10000 Zagreb, Croatia

**Keywords:** cytokine, alcoholic liver disease, single nucleotide polymorphism, liver transplantation, PNPLA3 148M, chemokine (C-C motif) ligand 2

## Abstract

*Background and Objectives*: Patients with GG rs738409 patatin-like phospholipase domain-containing protein 3 (PNPLA3) genotype (148M variant) have greater risk to develop end-stage liver disease and its associated clinical complications, including hepatocellular carcinoma (HCC). We aimed to analyze the association between the PNPLA3 genotype and augmented inflammatory response in transplant candidates with end-stage alcoholic liver disease (ALD). *Materials and Methods*: Concentrations of 13 cytokines were measured in 106 end-stage ALD patients without HCC (40 with CC, 40 with CG, and 26 with GG genotype), 35 end-stage ALD patients with HCC, and 19 control patients by cytometric bead array. *Results*: We found significantly higher concentrations of IL-1, IFN-α, IFN-γ, TNF-α, IL-6, CXCL8, IL-10, IL-12, IL-32, and IL-33 in patients with ALD compared to controls, while the concentration of CCL2 was significantly lower. No differences were observed in the concentration of IL-17 and IL-18. ALD patients with and without HCC had similar cytokine concentrations (*p* > 0.05 for all comparisons). End-stage ALD patients without HCC of the GG genotype had significantly higher CCL2 concentrations (212.6 [135.9–264.9] pg/mL) compared to end-stage ALD patients without HCC carrying the CC/CG genotypes (141.3 [104.1–201.6] pg/mL, *p* = 0.002, Mann–Whitney). No significant differences across the genotypes were found for the remaining measured cytokines (*p* > 0.05). GG carriers also had significantly higher levels of AST and ALT, and lower platelet counts. *Conclusions*: End-stage ALD patients without HCC who carry the PNPLA3 GG genotype have relatively higher CCL2 levels compared to those with the CC or CG genotypes. Relatively elevated CCL2 concentrations in GG patients might contribute to their increased risk of developing clinical complications compared to CC/CG patients.

## 1. Introduction

Excessive alcohol consumption leads to a significant number of diseases, with an estimated 2.07 million men and 0.374 million women dying from alcohol-related causes per year worldwide [1]. The development of end-stage liver disease is a common complication of alcohol use disorder and can only be treated through liver transplantation. Transplant candidates with end-stage alcoholic liver disease (ALD) are at greater risk for the occurrence of hepatocellular carcinoma (HCC) [2]. One of the primary genetic risk factors for developing liver-related complications of alcohol use disorder is the presence of the G allele in the rs738409 single nucleotide polymorphism (SNP) in the gene encoding patatin-like phospholipase domain-containing protein 3 (PNPLA3).

We have previously shown that Croatian patients with the GG rs738409 genotype have an increased risk of developing end-stage ALD as well as HCC [3,4]. Similar findings have been reported in other populations [5,6,7]. A meta-analysis conducted by Zhang et al. demonstrated an association between the G allele and HCC across various liver etiologies, with the strongest association observed for alcohol-related HCC and the weakest for HCV-related cirrhosis [8]. Furthermore, the progression of ALD, the time to decompensation, and the development of hepatic encephalopathy were shown to be faster in G allele carriers compared to those with the wild-type C allele. Mutation of this gene is also linked to shorter survival on the liver transplant list [9,10].

The PNPLA3 gene encodes the protein adiponutrin, an enzyme involved in various lipid metabolism processes. Although its exact roles are not fully understood, it is known to facilitate the hydrolysis of triacylglycerol, diacylglycerol, and monoacylglycerol, with a strong preference for oleic acid as the acyl moiety [11]. Carriers of the G allele have an isoleucine-to-methionine substitution at residue 148, which results in several alterations in lipid metabolism. For example, individuals with the GG genotype exhibit higher levels of very long-chain polyunsaturated fatty acids, a reduced ratio of saturated to polyunsaturated triacylglycerol in hepatocytes, and an increased content of retinyl esters in stellate cells, which is associated with lower plasma levels of retinols [12,13,14]. These lipid metabolic changes in G allele carriers might increase the risk of developing metabolic-associated liver disease (MASLD) [15]. Additionally, increased hepatic inflammation has been linked to the G allele variant of rs738409, though the precise molecular mechanisms remain unclear [16].

The inflammatory response plays a crucial role in the development of end-stage ALD and the occurrence of HCC. Various inflammatory cytokines have been implicated in liver fibrogenesis and carcinogenesis. For instance, tumor necrosis factor α (TNF-α) levels are elevated in end-stage ALD, and numerous studies suggest its potential contribution to carcinogenesis [17]. Additionally, increases in IL-6 and IL-10 levels have been observed in individuals with alcohol use disorder. Although these cytokines are thought to protect the liver from further damage, elevated IL-6 levels may also be associated with the development of carcinoma [18,19,20]. Degre et al. reported that plasma levels of chemokine (C-C motif) ligand 2 (CCL2) are increased in ALD and correlate with disease severity. Studies using animal and in vitro models further support the conclusion that CCL2 contributes to the growth and invasiveness of HCC [21,22,23].

Although an augmented inflammatory response has been implicated as a potential reason for the higher risk of clinical complications among G allele rs738409 carriers, data on its impact on cytokine expression remain scarce. Moreover, cytokines specifically associated with the risk allele in liver transplant candidates have yet to be identified. In the present study, we aimed to analyze the association between the PNPLA3 genotype and the concentration of 13 inflammatory cytokines in patients with end-stage ALD. Our findings reveal higher levels of CCL2 in end-stage ALD patients without HCC who carry the GG genotype when compared to those with the CC or CG genotype.

## 2. Materials and Methods

### 2.1. Patients and Study Design

Approval for the study was granted by the Ethics Committees of Merkur University Hospital (Case number: 0311-11070, date 7 December 2017) and the School of Medicine, University of Zagreb (Case number: 380-59-10106-18-111/251, date 13 December 2018). After obtaining written informed consent, 106 liver transplant candidates with end-stage ALD without HCC (end-stage ALD group), 35 end-stage ALD patients with HCC (HCC group), and 19 control patients without liver or inflammatory conditions were included in the study. All participants were of Caucasian ethnicity and had no concomitant liver etiologies (viral, autoimmune, or metabolic). Laboratory data, including alanine aminotransferase (ALT), aspartate aminotransferase (AST) activities, platelet count, international normalized ratio (INR), bilirubin, creatinine, and alpha-fetoprotein (AFP) levels on the day of study inclusion, were collected from the patients’ hospital clinical records.

For the group of end-stage ALD without HCC, from the initial pool of 200 genotyped patients, a total number of 26 patients with GG genotype were recruited into the study. From the remaining 174 patients with CC or CG genotype, we selected 40 age- and sex- matched patients with CC and CG genotype, respectively. The absence of HCC was confirmed on regular checkups by radiological findings and AFP concentration determination. For the patients who received liver transplant, HCC absence was further confirmed by the pathological examination of the explanted liver.

The primary goal of our study was to investigate differences in cytokine concentrations across various PNPLA3 genotypes in end-stage ALD patients without HCC. The control and HCC groups were included as references; however, comparisons between genotypes in these two groups were not part of the study design and are not presented in the results. The control group comprised 19 patients without liver, autoimmune, or chronic or acute infectious diseases. To avoid bias due to underrepresentation of the GG genotype in the control group, we included 5 patients with the GG genotype, 7 with the CC genotype, and 7 with the CG genotype from a previously genotyped cohort to match the genotype distribution seen in the ALD patient group [4]. The HCC group consisted of 35 patients with HCC developed in end-stage ALD, confirmed by radiological imaging and liver biopsy.

After obtaining written informed consent to participate in the study, 5 mL of venous blood samples were taken in the morning (between 7 and 9 a.m.), 200 µL of whole blood was stored at −20 °C until DNA isolation and genotype analysis, and plasma was obtained by centrifugation and stored at −80 °C until analysis.

### 2.2. DNA Isolation and Genotyping

DNA was extracted from 200 µL of whole blood samples using the QIAGEN QIAamp DNA Blood Mini Kit (Qiagen, Venlo, The Netherlands, Cat. No. 51106) spin method, following the manufacturer’s instructions as described previously [4]. DNA concentration and quality were assessed using the NanoDrop ND1000 spectrophotometer (Thermo Fisher Scientific, Waltham, MA, USA). All samples were stored at −20 °C until genotype analysis. Genotypes were determined by polymerase chain reaction (PCR) using the commercially available TaqMan SNP assay for PNPLA3 rs738409, which employs two fluorescent dyes—FAM and VIC—to differentiate between alleles (assay ID: C______7241_10, Cat. No. 4351379). PCR amplification was carried out using the ABI 7500 instrument (Applied Biosystems, Foster City, CA, USA).

### 2.3. Cytometric Bead Array

Concentrations of IL-1β, IL-6, chemokine (C-X-C motif) ligand 8 (CXCL8), IL-10, IL-12, IL-17, IL18, IL-32, IL-33, CCL2, IFNα, IFN-γ, TNF-α were measured by cytometric bead array-based technique using the LEGENDplex™ Human Inflammation Panel 1 (13-plex) with Filter Plate (Cat. No. 740808, Biolegend, San Diego, CA, USA) according to the manufacturer’s instructions using FACSAria II (BD Biosciences, Franklin Lakes, NJ, USA) instrument as described previously [24]. Briefly, samples were diluted 1:2 in dilution buffer and incubated with capture beads. Each bead was coated with a specific capture antibody for each soluble protein and could be identified by its unique combination of bead physical properties (FSC-SSC) and fluorescence (APC) signature on the flow cytometer. After washing, the samples were incubated with the detection reagent and washed again. The complexes of capture beads, analytes, and detection reagents were analyzed using the FACSAria II instrument (BD Biosciences, Franklin Lakes, NJ, USA). Data analysis was performed using the LEGENDplex™ Data Analysis Software Suite version 2023-11-07, available on the manufacturer’s website.

### 2.4. Statistical Analysis

Data are presented as median with interquartile range and were analyzed using the Mann–Whitney test or the Kruskal–Wallis test, followed by the Mann–Whitney test with Holm–Bonferroni correction. Effect size r was calculated by dividing the z-statistic by the square root of the total sample size. Spearman’s rank correlation coefficient was calculated for correlations. The chi-square test was used to compare categorical data. All tests were two-tailed, with *p* < 0.05 considered statistically significant. Data analysis was performed using GraphPad Prism version 6 for Windows (GraphPad Software Inc., San Diego, CA, USA).

## 3. Results

Demographic data of the patients are shown in Table 1. There was no significant difference in age between the end-stage ALD group without HCC (60 [55–65] years), the HCC group (60.5 [55–65] years), and the control group (58 [51–71] years) (*p* = 0.57, Kruskal–Wallis). However, the HCC group had a significantly higher proportion of male patients (97.1%) compared to the other two groups (83% in the end-stage ALD group and 73.7% in the control group). In accordance with the study design, there were no significant differences in the percentage of GG genotype carriers among the groups: 26.3% in the control group, 24.5% in the end-stage ALD without HCC group, and 22.9% in the HCC group.

### 3.1. Concentration of Acute Proinflammatory Cytokines in End-Stage Alcoholic Liver Disease

**Figure 1:**
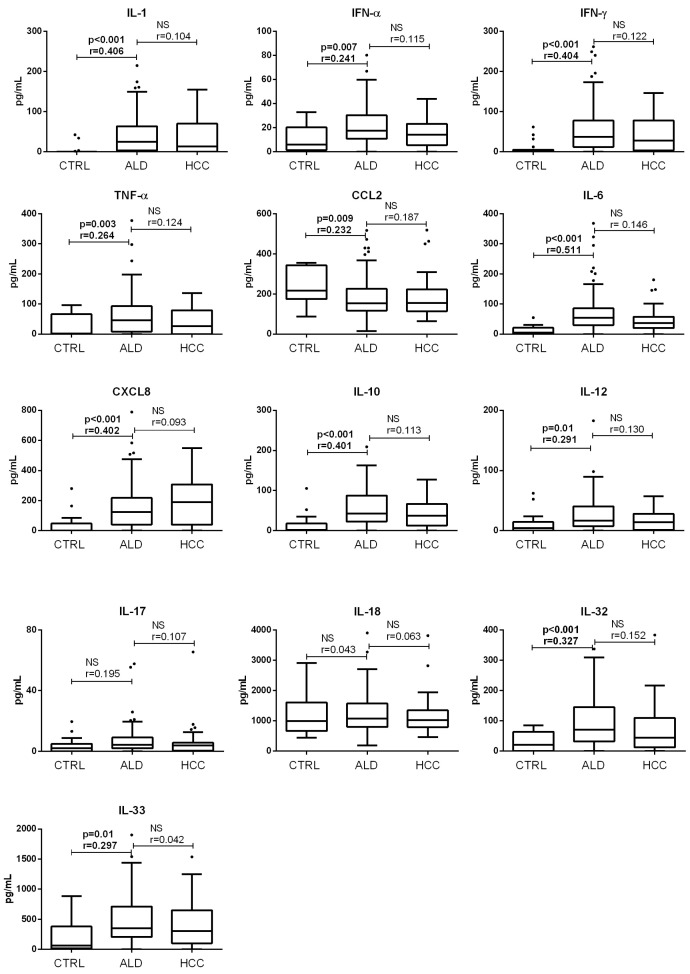
Concentrations of inflammatory cytokines in control, end-stage ALD and HCC groups of patients. Concentrations of cytokines were determined by flow cytometry. Box and whiskers plot represent median (central line) with interquartile range (values that fall more than 1.5 times the interquartile range above the upper quartile or below the lower quartile). Comparisons were made by Kruskal–Wallis test followed by Mann–Whitney (*n* = 19, 106 and 35, for control, end-stage ALD, and HCC group, respectively); effect size (r) was calculated by dividing the z-statistic by the square root of the sample size. NS—non significant. ALD—alcoholic liver disease; CCL2—chemokine (C-C motif) ligand 2; CTRL—control group; CXCL8—chemokine (C-X-C motif) ligand; HCC—hepatocellular carcinoma; IFN—interferon; IL—interleukin; TNF tumor necrosis factor.

Concentrations of the analyzed cytokines in the control, end-stage ALD without HCC, and HCC groups are shown in Figure 1. For ten of the thirteen cytokines analyzed (IL-1, IFN-α, IFN-γ, TNF-α, IL-6, CXCL8, IL-10, IL-12, IL-32, and IL-33), significantly higher concentrations were found in patients with end-stage ALD without HCC compared to controls, confirming the modulation of immune response in end-stage ALD without HCC. Unexpectedly, CCL2 concentrations were significantly lower in patients with end-stage ALD (153.6 [117.9–225] pg/mL, median with interquartile range) compared to controls (217.9 [174.7–343.2] pg/mL, *p* < 0.01, Kruskal–Wallis followed by Mann–Whitney). The concentrations of IL-17 and IL-18 were similar in both groups. The HCC group of patients did not significantly differ from patients with end-stage ALD without HCC in terms of CCL2 concentration (155.1 [114.2–223.4] pg/mL, *p* > 0.05) or in any of the remaining 12 cytokines (*p* > 0.05 for all analyses).

**Figure 2:**
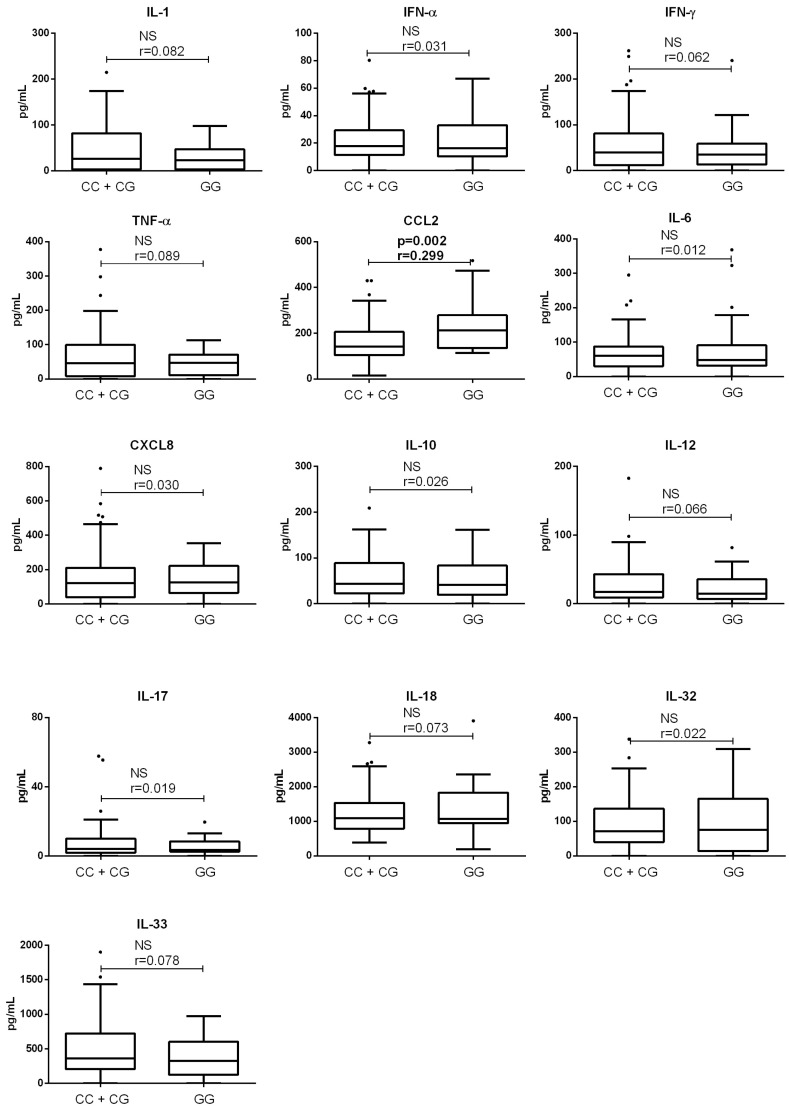
Comparison of proinflammatory cytokine concentrations in end-stage ALD patients without HCC between the patients with CC/CG and GG rs738409 PNPLA3 genotype. Concentrations of cytokines were determined by flow cytometry; genotypes were determined by PCR. Box and whiskers plot represent median (central line) with interquartile range, dots show outliers (values that fall more than 1.5 times the interquartile range above the upper quartile or below the lower quartile). Comparisons were made by Mann–Whitney (*n* = 80, and 26, for CC/CG and GG genotype, respectively); effect size (r) was calculated by dividing the z-statistic by the square root of the sample size. NS—non significant. ALD—alcoholic liver disease; CCL2—chemokine (C-C motif) ligand 2; CXCL8—chemokine (C-X-C motif) ligand 8; IFN—interferon; IL—interleukin; TNF—tumor necrosis factor.

There was no significant difference in age or sex between end-stage ALD patients without HCC categorized by PNPLA3 genotype (Table 2). Analysis of plasma cytokine concentrations in patients with end-stage ALD without HCC (GG genotype vs. CC/CG genotype) revealed that GG carriers had significantly higher concentrations of CCL2 (212.6 [135.9–264.9] pg/mL) compared to CC/CG carriers (141.3 [104.1–201.6] pg/mL, *p* = 0.002, Mann–Whitney). No significant differences were found for the remaining 12 cytokines (*p* > 0.05 for all analyses) (Figure 2).

**Figure 3:**
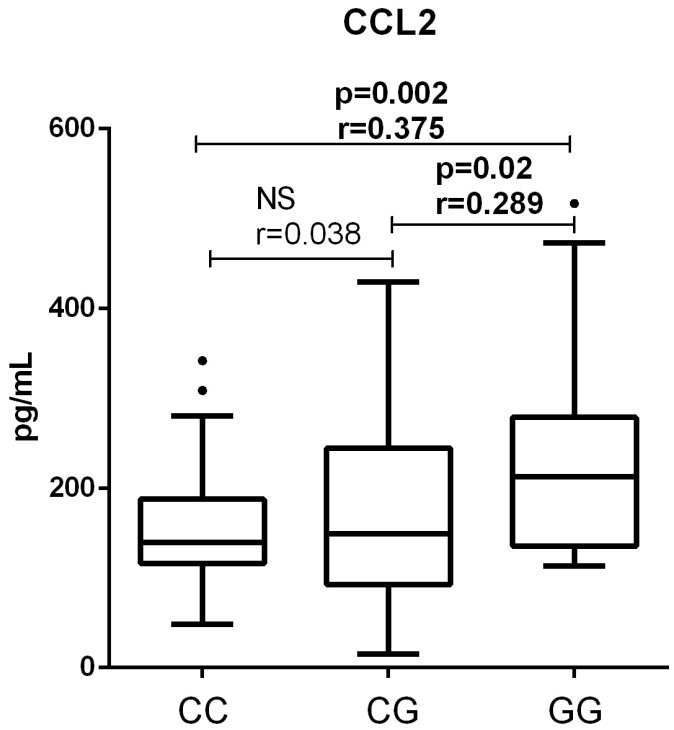
Comparison of CCL2 concentrations in end-stage ALD patients without HCC between the patients with CC, CG, and GG rs738409 PNPLA3 genotypes. Concentrations of CCL2 were determined by flow cytometry; genotypes were determined by PCR. Box and whiskers plot represent median (central line) with interquartile range, dots show outliers (values that fall more than 1.5 times the interquartile range above the upper quartile or below the lower quartile). Comparisons were made by Kruskal–Wallis test followed by Mann–Whitney (*n* = 40, 40 and 26, for CC, CG, and GG genotype, respectively); effect size (r) was calculated by dividing the z-statistic by the square root of the sample size. NS—non significant. CCL2—chemokine (C-C motif) ligand 2.

Comparison of CCL2 concentrations across the all three genotypes in end-stage ALD patients without HCC revealed a significant difference (*p* < 0.001, Kruskal–Wallis test). Post hoc analysis showed that end-stage ALD patients with the GG genotype had significantly higher CCL2 levels than those with the CC (*p* = 0.002, effect size r = 0.375) or CG genotype (*p* = 0.02, effect size r = 0.289). However, no significant difference was observed between CC (139.1 [117.0–185.7] pg/mL) and CG carriers (149.3 [93.0–238.1] pg/mL) (*p* > 0.05, effect size r = 0.038, post hoc Mann–Whitney test, Figure 3). No significant differences were observed for any of the other 12 cytokines in these comparisons (see Supporting Information, Figure S1).

### 3.2. Association of PNPLA3 Genotype with Clinical Parameters of Patients with End-Stage ALD

**Figure 4:**
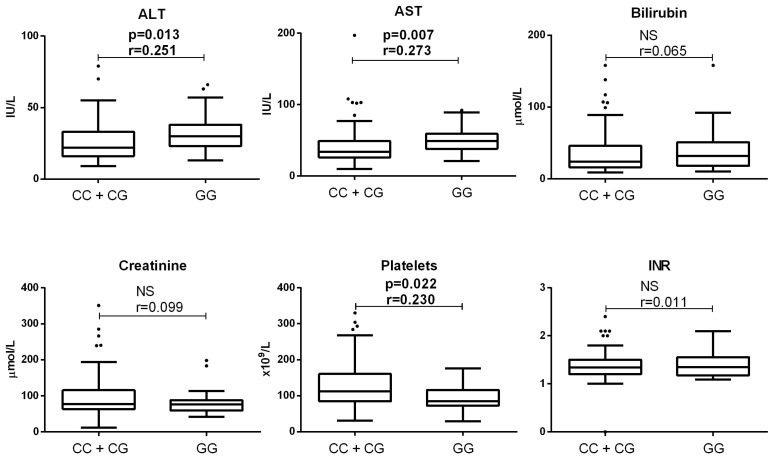
Comparison of clinical parameters in end-stage ALD patients without HCC between the patients with CC/CG and GG rs738409 PNPLA3 genotype. Findings were collected from patient medical records and genotypes were determined by PCR. Box and whiskers plot represent median (central line) with interquartile range; dots show outliers (values that fall more than 1.5 times the interquartile range above the upper quartile or below the lower quartile). Comparisons were made by Mann–Whitney (*n* = 80, and 26, for CC/CG and GG genotype, respectively); effect size (r) was calculated by dividing the z-statistic by the square root of the sample size. NS—non significant, ALT—alanine aminotransferase; AST—aspartate transaminase; INR—international normalized ratio.

End-stage ALD patients with the GG genotype had significantly higher plasma ALT (30 (23–38) vs. 16 (22–33), *p* = 0.013, Mann–Whitney test) and AST (49 (38–59) vs. 34 (26–49), *p* = 0.007, Mann–Whitney test) activities compared to patients with the CC/CG genotypes. Additionally, platelet count was significantly lower in patients with the GG genotype (85.0 (72–116)) compared to those with the CC/CG genotypes (112.5 (85.5–159), *p* = 0.022, Mann–Whitney test). No significant differences were found in bilirubin, creatinine, or INR between genotypes (*p* > 0.05, Figure 4).

**Figure 5:**
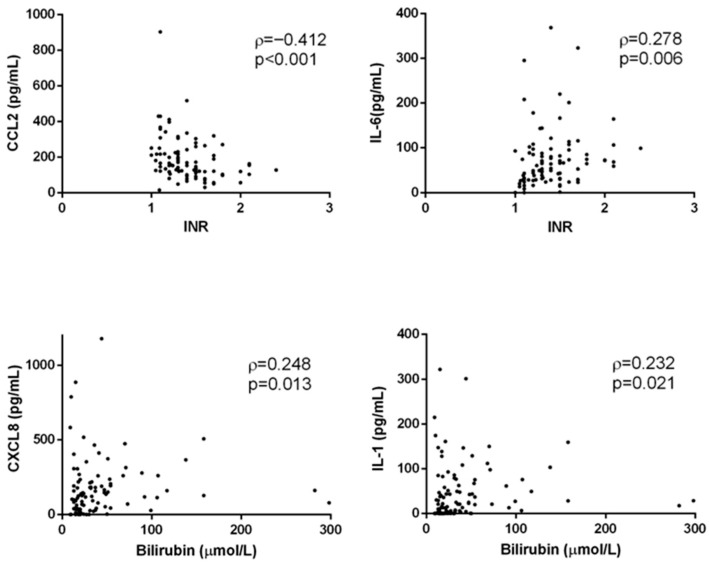
Selected correlations between concentrations of cytokines and clinical parameters of patients. Concentrations of cytokines were determined by flow cytometry; laboratory data were collected from medical records of patients. Dots represent individual values (*n* = 106). CCL2—chemokine (C-C motif) ligand 2; CXCL8—chemokine (C-X-C motif) ligand 8; INR—international normalized ratio; ρ—Spearman coefficient.

**Figure 6:**
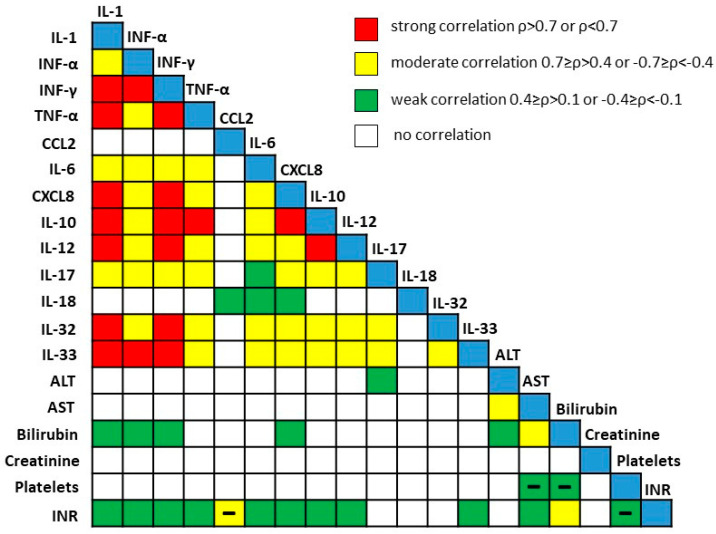
Correlation between the concentration of cytokines and clinical findings of patients with end-stage ALD. Concentrations of cytokines were determined by flow cytometry; laboratory data were collected from medical records of patients. Red squares represent strong correlation with Spearman coefficient >0.7 or <−0.7; yellow squares represent moderate correlations with Spearman coefficient <0.7 and >0.4 or <−0.7 and >−0.4; green squares represent weak correlations with Spearman coefficient <0.4 and >0.1 or <−0.1 and >−0.4. White squares represent no significant correlation. Empty squares denote a positive Spearman coefficient, while minus sign denotes negative correlation. ALT—alanine aminotransferase; AST—aspartate transaminase; CCL2—chemokine (C-C motif) ligand 2; CXCL8—chemokine (C-X-C motif) ligand 8; IFN—interferon; IL—Interleukin, INR—international normalized ratio; TNF—tumor necrosis factor.

The correlation between the concentrations of the analyzed cytokines and clinical parameters is shown in Figure 5. A significant but generally moderate to weak correlation was found only for a few cytokine–clinical parameter pairs. For example, CCL2 concentrations showed a negative correlation with INR (ρ = −0.412, *p* < 0.001), while IL-6 showed a weaker, positive correlation with INR (ρ = 0.278, *p* < 0.01). CXCL8 (ρ = 0.248, *p* < 0.05) and IL-1 (ρ = 0.232, *p* < 0.05) concentrations correlated with bilirubin levels. On the other hand, acute inflammatory cytokines showed strong correlations with each other, with the strongest correlation observed between IL-1 and IFN-γ concentrations (ρ = 0.935, *p* < 0.001, Figure 6).

## 4. Discussion

The association between the PNPLA3 rs738409 SNP and the risk of further complications in individuals with end-stage ALD is well-established through numerous studies and meta-analyses. The loss of function mutation affects lipid metabolism, resulting in abnormal concentrations of fatty acids and retinyl esters in the livers of G allele carriers [3,8,14]. These alterations are thought to trigger an inflammatory response, potentially increasing the risk of HCC development. However, the precise molecular mechanisms remain unclear. In the current study, we identified an association between CCL2 concentration and the GG genotype in end-stage ALD patients without HCC. We hypothesize that relatively higher concentrations of CCL2 in end-stage ALD patients with the GG genotype than in the end-stage ALD patients with the CC/CG genotype may contribute to their greater risk of developing clinical complications.

Previous studies have shown that visceral adipose tissue secretes CCL2 in obesity, and that CCL2 levels decrease with weight loss [25,26]. Given that the PNPLA3 G allele is associated with increased lipid accumulation, our findings align with these observations. However, we were surprised to find significantly lower levels of CCL2 in patients with end-stage ALD compared to the control group. This contrasts with previous studies reporting elevated CCL2 levels in conditions like alcoholic hepatitis and fibrosis associated with primary biliary cholangitis [27,28]. It is important to note that our patients had end-stage ALD, where normal liver tissue is almost completely replaced by fibrotic tissue, which may be less active in CCL2 production. Supporting this notion, Ferrari-Cestari et al. recently demonstrated that CCL2 production in MASLD is more strongly associated with the degree of visceral adiposity rather than fibrosis [29]. Therefore, we speculate that the decrease in CCL2 observed in our patients with end-stage ALD may be due to cirrhosis-related fat loss, and that this reduction might be less pronounced in GG carriers due to impaired lipid metabolism. Further research involving patients at different stages of fibrosis is needed to test this hypothesis. Furthermore, we have explored GEO Datasets and found two relatable datasets, both of which indicate lower CCL2 in ALD. The most relevant one being GSE167308, where the CCL2 gene expression is higher in control liver tissue samples (healthy liver tissue fragments from tumor resections, *n* = 5, median differential gene expression (DGE) = 5) than in ALD (immediately following explantation of diseased liver, *n* = 7, median DGE = 1) [30]. Similarly, GSE141100 compares hepatic stellate cells (HSCs) from control (*n* = 8) and ALD samples (*n* = 6), again demonstrating lower CCL2 expression in the ALD group [31]. While these findings are consistent with our results, they reflect tissue-specific gene expression and thus cannot directly confirm changes in plasma CCL2 concentrations in ALD.

Our findings, which link the high-risk GG rs738409 genotype for HCC development with relatively elevated CCL2 levels, prompted us to review the literature for studies exploring the pathogenic role of CCL2 in liver carcinogenesis. Bartneck et al. reported that CCL2 inhibition in a fibrosis-HCC murine model significantly reduced pathogenic vascularization and tumor volume, while Li et al. observed similar results with a CCR2 antagonist [32,33]. These findings suggest that the increased CCL2 levels observed in GG carriers may contribute to the development of carcinogenesis. However, as CCL2 appears to be only a contributory factor to the carcinogenesis, we believe that in the absence of carcinogenic conditions such as end-stage ALD, its higher concentrations are not per se enough to facilitate HCC development. Regarding our results, a higher concentration of CCL2 in healthy controls may not influence the risk of HCC development, as there is no predisposing liver condition. In contrast, the relatively higher CCL2 levels observed in end-stage ALD patients with the GG genotype could increase the risk of HCC compared to those with the CC/CG genotypes. We did not observe an increase in CCL2 in HCC patients, but this does not rule out its contributory role during carcinoma development. Further research is needed to test a possible mechanistic role of CCL2 in the increased risk of GG patients to develop HCC or other clinical complications. If future research confirms our findings, CCL2 could potentially serve as a biomarker or a personalized therapeutic target for GG patients. Notably, drugs targeting the CCL2/CCR2 axis are undergoing pre-clinical and clinical trials for various conditions [34,35].

Increased levels of IL-1, IL-6, CXCL-8, IL-10, IL-12, IL-32, IL-33, IFN-α, IFN-γ, and TNF-α in end-stage ALD compared to controls suggest the presence of an inflammatory response in this condition. However, these cytokine concentrations were not associated with the PNPLA3 rs738409 genotype, indicating that they are not linked to an increased risk of clinical complications in GG carriers with end-stage ALD. As *p* values are far away from the significance threshold with a small effect size, there is no indication that some of the differences would be detected with larger sample sizes. It is important to note, however, that this does not rule out the possibility of earlier alterations in these cytokines in GG carriers, which may contribute to the risk of fibrogenesis. To explore this, similar investigations in patients at earlier stages of fibrosis are needed. Overall, cytokine concentrations did not correlate strongly with biochemical markers of liver disease, though it is possible that a stronger correlation could be observed in earlier stages of liver disease.

For the ten cytokines found to be elevated in end-stage ALD, we did not observe a further increase in HCC. The existing literature indicates that inflammatory cytokine concentrations are elevated in cirrhosis; however, data on whether these levels increase further in HCC are variable, likely depending on the underlying etiology of the disease [36,37]. Even though, there are similarities in the regulation of CCL2 and the cytokines that were elevated in end-stage ALD patients, the cellular sources of these cytokines and mechanism of secretion differ, and the observed differences in concentration may arise from variations in cell stimulation [38,39].

We found one similar study in the literature, conducted by Peregud et al., that attempted to associate the rs738409 genotype with cytokine levels in ALD. Their study reported no significant association with CCL2 or other analyzed cytokines [40]. However, our study design offered several advantages that enhanced its power. For instance, patients with end-stage ALD were on the transplant list, ensuring regular check-ups and alcohol abstinence. Additionally, our genotyped patient pool allowed us to select age- and sex-matched individuals across genotypes and include a larger number of GG carriers. In contrast, the study by Peregud et al. had a relatively small number of GG carriers, which likely explains why only a GG/CG vs. CC comparison was made, showing no significant difference. However, they did observe moderately higher CCL2 levels in GG/CG carriers, which aligns with our findings. While their study found significantly higher CCL2 concentrations in end-stage ALD compared to controls, the results are not directly comparable to ours, as their control group consisted of individuals who were 10 years younger and had a history of alcohol abuse [40]. In a more recent study, Kirchmeyer et al. investigated the association between the PNPLA3 genotype and cytokine levels in MASLD and found no differences. One possible explanation for their negative results could be differences in the pathogenesis of ALD and MASLD [41]. Another possibility is that the sample size of GG genotype carriers in their study was too small, with only 11 patients with MASLD carrying the GG genotype [42].

### Limitations

Our study has several limitations that should be carefully considered. Like other studies, a key limiting factor for the study’s power was the relatively low number of patients with the GG genotype. However, the availability of a larger genotyped cohort allowed us to mitigate this limitation by including all available GG carriers. The study’s power was further enhanced by selecting a greater number of age- and sex-matched patients with CC and CG genotypes. We acknowledge, as a study limitation, that a larger sample size could potentially reveal additional differences in cytokine levels. However, the clinical relevance of such findings would likely be limited, given that the largest effect size among the non-significant results across the genotypes was only 0.089. Analysis of the calculated effect sizes for any comparison further indicates that any effect size greater than 0.2 was associated with a statistically significant *p* value, supporting the conclusion that the study had sufficient power to detect relevant associations.

It is also important to note that possible confounding could arise from differences in body mass index, alcohol abstinence duration, or comorbidities. Finally, although our results suggest that higher levels of CCL2 in high-risk patients may play a contributory role in the development of clinical complications such as HCC, further mechanistic research is required to validate this hypothesis.

## 5. Conclusions

End-stage ALD patients without HCC who carry the high-risk GG PNPLA3 genotype have relatively higher CCL2 levels compared to those with the CC or CG genotypes. Elevated CCL2 concentrations in GG patients might contribute to their increased risk of developing clinical complications compared to CC/CG patients. The potential of CCL2 as a biomarker or therapeutic target remains to be investigated.

## Figures and Tables

**Table 1:** Demographic data and distribution of genotypes.

	Control	ALD	HCC	*p* Value
*n*	19	106	35	-
Age ^1^	58 (51–71)	60 (55–65)	60.5 (55–65)	0.57
Sex ^2^				0.04
female	5 (26.3%)	18 (17%)	1 (2.9%)	
male	14 (73.7%)	88 (83%)	34 (97.1%)	
rs738409 PNPLA3 genotype ^2^				
CC	7 (36.8%)	40 (37.7%)	10 (28.6%)	0.82
CG	7 (36.8%)	40 (37.7%)	17 (48.6%)	
GG	5 (26.3%)	26 (24.5%)	8 (22.9%)	

^1^ For age, median and interquartile range are shown, and *p* value was calculated by Kruskal–Wallis test. ^2^ For sex and genotype, number of patients with percentage are shown, and *p* value was calculated by chi-square test. ALD—group of end-stage alcoholic liver disease patients without HCC; HCC—hepatocellular carcinoma, PNPLA3—patatin-like phospholipase domain-containing 3.

**Table 2:** Demographic data of end-stage ALD patients without HCC by PNPLA3 genotypes.

	CC/CG	GG	*p*	CC	CG	GG	*p*
*n*	80	26		40	40	26	-
Age ^1^, years	60.5 (55–65)	58 (55–63)	0.45	61 (55–65)	59.5 (56–64.5)	58 (55–63)	0.75
Sex ^2^			0.40				0.67
female	15 (18.8%)	3 (11.5%)		8 (20%)	7 (17.5%)	3 (11.5%)	
male	65 (81.3%)	23 (88.5%)		32 (80%)	33 (82.5%)	23 (88.5%)	

^1^ For age, median and interquartile range are shown, and *p* value was calculated by Kruskal–Wallis test. ^2^ For sex, number of patients with percentage are shown and *p* value was calculated by chi-square test.

## Data Availability

Data are contained within the article or Supplementary Material. Any additional data are available from the corresponding author upon reasonable request.

## Supplementary Materials

The following supporting information can be downloaded at https://www.mdpi.com/article/10.3390/medicina61071293/s1, Figure S1 Comparison of cytokine concentrations in end-stage ALD between patients with CC, CG and GG rs738409 PNPLA3 genotypes.

## Abbreviations

The following abbreviations are used in this manuscript:

AFP	alpha-fetoprotein
ALT	alanine aminotransferase
AST	aspartate aminotransferase
ALD	alcoholic liver disease
CCL2	chemokine (C-C motif) ligand 2
CTRL	control group
CXCL8	chemokine (C-X-C motif) ligand 8
HCC	hepatocellular carcinoma
IFN	interferon
IL	interleukin
PNPLA3	patatin-like phospholipase domain-containing protein 3
TNF-α	tumor necrosis factor α
