# Supplementary material for: High-Risk PNPLA3 rs738409 Genotype Is Associated with Higher Concentrations of CCL2 in Liver Transplant Candidates with Alcoholic End-Stage Liver Disease
Source: Medicina (Kaunas). 2025 Jul 18;61(7):1293. doi: 10.3390/medicina61071293 (PMC12300434; doi:10.3390/medicina61071293)
Supplement: Supplementary file 1 [file medicina-61-01293-s001.zip › medicina-3713277-supplementary.pdf]

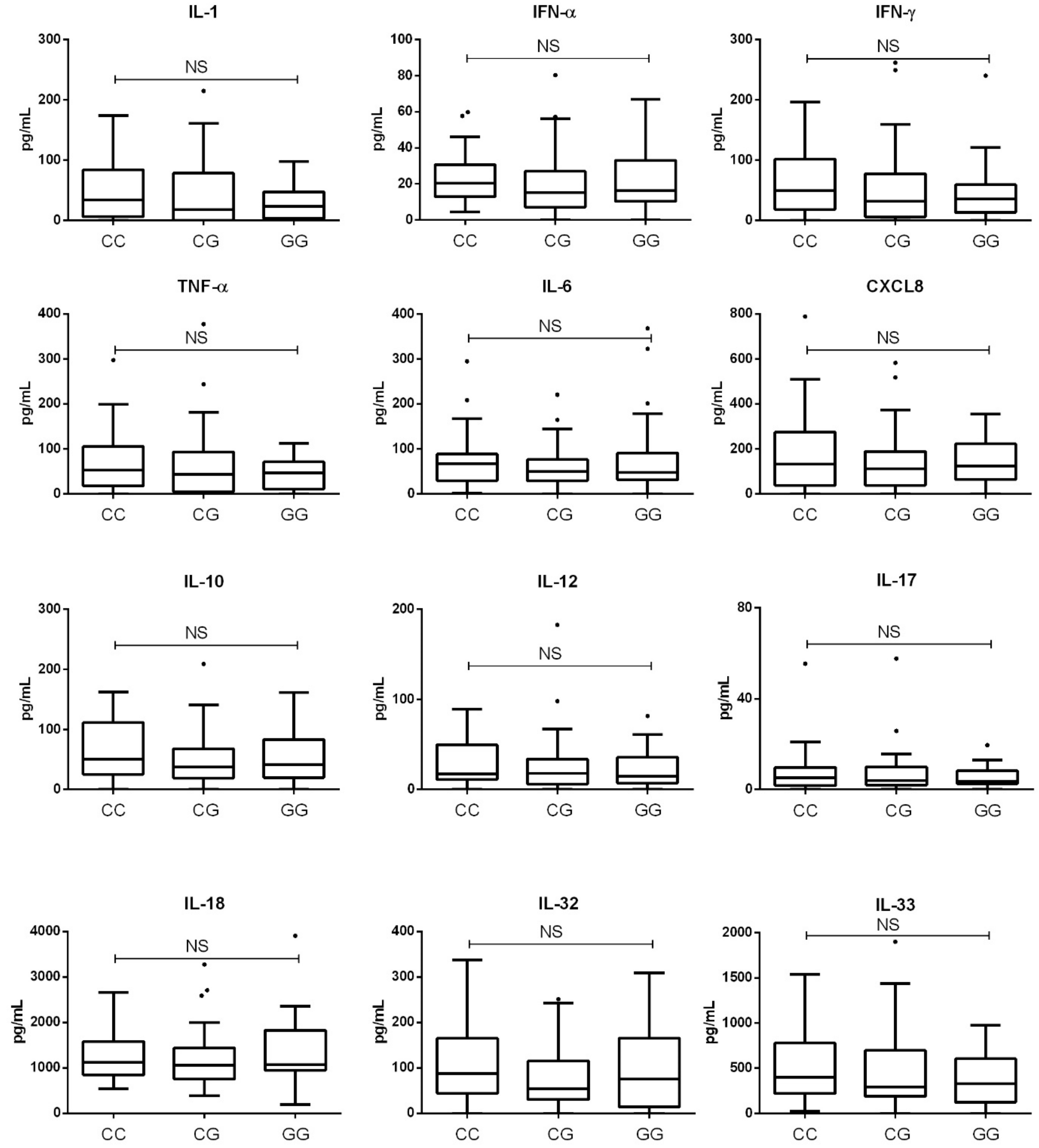

Figure S1 Comparison of cytokine concentrations in end-stage ALD between patients with CC, CG and GG rs738409 PNPLA3 genotypes. Concentrations of cytokines were determined by flow cytometry, genotypes were determined by PCR. Box and whiskers plot represent median with interquartile range, dots show outlier values. Comparison were made by Kruskal-Wallis test (N= 40, 40 and 26, for CC, CG and GG genotype, respectively). NS – non significant

CXCL8 – chemokine (C-X-C motif) ligand; IFN –interferon; IL – Interleukin, TNF – tumor necrosis factor
